# Microbial Monitoring of Crewed Habitats in Space—Current Status and Future Perspectives

**DOI:** 10.1264/jsme2.ME14031

**Published:** 2014-08-12

**Authors:** Nobuyasu Yamaguchi, Michael Roberts, Sarah Castro, Cherie Oubre, Koichi Makimura, Natalie Leys, Elisabeth Grohmann, Takashi Sugita, Tomoaki Ichijo, Masao Nasu

**Affiliations:** 1 Environmental Science and Microbiology, Graduate School of Pharmaceutical Sciences, Osaka University, 1–6, Yamadaoka, Suita, Osaka 565–0871, Japan; 2 Space Life Sciences Lab, CASIS, NASA Kennedy Space Center, FL 32899, USA; 3 Biomedical Research and Environmental Sciences Division, NASA Johnson Space Center, Houston, TX 77058, USA; 4 Science, Technology & Engineering Group, Wyle, 1290 Hercules Avenue, Houston, Texas 77058, USA; 5 Laboratory of Space and Environmental Medicine, Graduate School of Medicine, Teikyo University, 359, Otsuka, Hachioji, Tokyo 192–0395, Japan; 6 Molecular and Cellular Microbiology, Belgian Nuclear Research Center SCK-CEN, Boertang 200, BE-2400, Mol, Belgium; 7 Division of Infectious Diseases, University Medical Centre Freiburg, Hugstetter Strasse 55, D-79106 Freiburg, Germany; 8 Department of Microbiology, Meiji Pharmaceutical University, 2–522–1 Noshio, Kiyose, Tokyo 204–8588, Japan

**Keywords:** International Space Station, microbial monitoring, on-site analysis

## Abstract

Previous space research conducted during short-term flight experiments and long-term environmental monitoring on board orbiting space stations suggests that the relationship between humans and microbes is altered in the crewed habitat in space. Both human physiology and microbial communities adapt to spaceflight. Microbial monitoring is critical to crew safety in long-duration space habitation and the sustained operation of life support systems on space transit vehicles, space stations, and surface habitats. To address this critical need, space agencies including NASA (National Aeronautics and Space Administration), ESA (European Space Agency), and JAXA (Japan Aerospace Exploration Agency) are working together to develop and implement specific measures to monitor, control, and counteract biological contamination in closed-environment systems. In this review, the current status of microbial monitoring conducted in the International Space Station (ISS) as well as the results of recent microbial spaceflight experiments have been summarized and future perspectives are discussed.

Previous space experiments conducted during short-term flights and aboard orbiting space stations suggest that the relationship between humans and microbes may be changed in space habitats (environmentally controlled closed eco-systems under microgravity and elevated radiation). The pathogenicity and virulence of some bacteria, such as *Salmonella enterica* serovar Typhimurium, have been shown to increase under microgravity, and this has been attributed to enhancements in the formation of extracellular matrices and production of biofilms (59). The immune system responses of astronauts during spaceflight are also altered (6) possibly due to the stress associated with crewed habitats in space. Manned missions to Mars, which may be realized within the next two decades, may weaken the immune status of the crew due to long-duration missions in a confined environment and ultimately cause profound changes in the composition of the bacterial flora in the intestinal tract, nasal passages, and upper airways, resulting in an increased risk of infection. Therefore, research on the relationship between human physiology and microbial ecology in space habitats is critical for the longterm operation and sustaining engineering of a human presence in space (38). As outlined in the mission roadmaps of the international partners on the ISS, including NASA (National Aeronautics and Space Administration; Human Health, Life Support and Habitation Systems—Technology Area 06. http://www.nasa.gov/pdf/500436main_TA06-HHLSHS-DRAFT-Nov2010-A.pdf), ESA (European Space Agency; Towards Human Exploration of Space: a European Strategy [THESEUS] Roadmap. http://theseus.hd20.hosting.punkt.de/fileadmin/Docs/Eg_reports_roadmap/RoadMap_web.pdf), and JAXA (Japan Aerospace Exploration Agency; Kibo Utilization Scenario toward 2020 in the field of Life Science. http://iss.jaxa.jp/en/kiboexp/scenario/pdf/life%20science.pdf), rapid and robust environmental monitoring is required to provide essential information on microbiological safety (*e.g.*, define correct upper and lower thresholds of microbes in the air, surface, and water) to enable continued success in long-duration space habitation.

Additional research is needed to better understand the dynamics of microbial colonization under space habitat conditions and how interactions between microbial communities, humans, and life support systems impact on both human and system performance. Contamination by microorganisms in confined environments represents a potential risk for the health of the crew as well as on-board systems (37, 53). Therefore, each space agency has developed and implemented specific measures to prevent contamination by microorganisms, continuously monitor the crewed habitat and life support systems, and develop deployable microbial control and disinfection.

In this review, we describe the current status of microbial monitoring conducted on the ISS, the results of recent microbial spaceflight experiments, and future perspectives on international cooperation to share, standardize, and improve methods, procedures, and technology.

## Sampling in space habitats

**Fig. 1:**
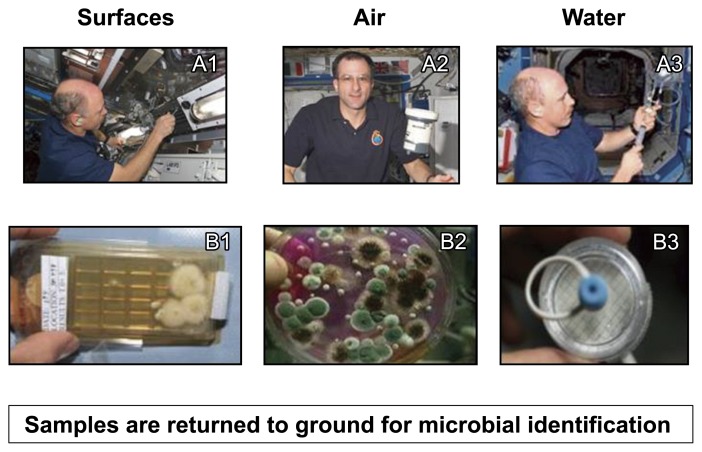
Spaceflight surface, air, and water samples are collected and microbial colonies are enumerated during flight, while microbial identification is performed on the ground. Inflight sample collection activities include swabbing surfaces (A1), air sample collection using air sample equipment (A2), and collecting water from potable water sources (A3). Enumeration is performed on incubated samples that include contact slides from surface samples (B1), culture plates from air samples (B2), and colony growth on filter discs from water samples (B3).

NASA has monitored the presence of microorganisms since early human spaceflight into a low Earth orbit (an altitude between 160 km and 2,000 km) including the NASA-Mir and Space Shuttle programs. Preflight surface, air, and water samples are typically collected between 2 months to 10 d prior to a launch based on the sample type. Samples include surface swabs of randomly selected hardware items as well as air and surface samples from the habitable volume of the vehicle. Air, surface, and water samples are also collected on a quarterly basis with microbial enumeration being performed during flight and microbial identification on samples returned to the ground for analysis (Fig. 1, Table 1).

**Fig. 2:**
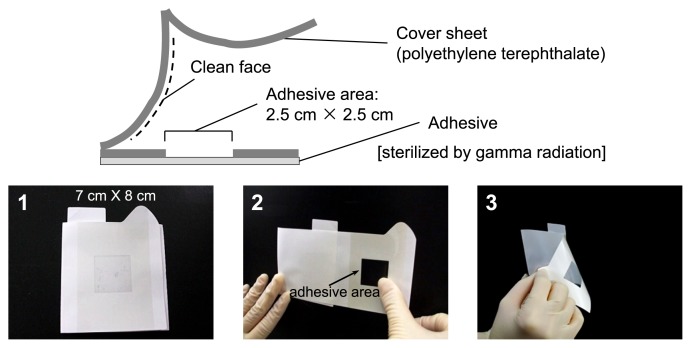
Adhesive sheet for microbial monitoring in the space habitat. 1. Photograph of the adhesive sheet; 2. Attach the adhesive area to the sampling site and press; 3. peel the adhesive sheet off the sampling site.

A swab is widely used for ground processing and on-orbit surface sampling of space vehicles (61); however, this approach requires water to wet the surface for effective microbial collection, and multiple steps are involved in the current swab sampling protocol. Therefore, new sampling devices with simplified procedures are needed. One such device uses an adhesive sheet with several improvements over swab-based methods, including a simplified procedure, no water requirement for sample collection, and improved ease of transport and storage (Fig. 2). The ability of the adhesive sheet to collect microbes from a metal plate and laptop palm rest (plastic, rough surface) was found to be equivalent to that of the swab (24). Therefore, the adhesive sheet represents an alternative device for sampling in a space habitat and has been used for surface microbial sampling in Kibo, the Japanese Experiment Module of the ISS (24).

## Microbial bio-contamination control

NASA has obtained much information on the risks of microbial contamination to the crew members as well as the vehicle from spaceflight microbial environmental monitoring, and has consequently developed engineering controls and monitoring strategies to minimize detrimental microbial growth during spaceflight (39). The microbial control efforts of NASA are focused on preventing microbial growth by reducing humidity and eliminating free water, maintaining high-volume exchange and air filtration, and implementing a schedule of routine housekeeping and food monitoring. The microbial environmental acceptability limits for preflight and inflight (Table 2) were developed through spaceflight experience and data collected by subject matter experts in microbial monitoring and control. These limits have focused on crew health and safety in addition to the prevention of biofouling to maintain space vehicle system integrity.

If preflight or inflight samples exceed the defined limits, remediation or mitigation steps are implemented and include repeat sample collection to verify that remediation procedures are adequate to meet environmental specifications. An example of an inflight anomaly occurred in 2004 during Expedition 9 when a suspected mold was identified on a panel front in the Functional Cargo Block (Russian Segment on ISS). It was later discovered that wet clothing and towels were placed against the fabric panel to dry, causing a wet area that allowed for microbial growth. Initial remediation efforts included altering crew hygiene practices to prevent wet items from being placed in direct contact with the fabric surface and decrease the potential for microbial growth.

The prevention of bio-contamination should include rational habitat designs. For example, habitats designed to minimize the spread of biological aerosols benefit from the development of a reliable model to disperse bio-aerosol contamination from point or diffuse sources in the habitat. BIOSMHARS (BIOcontamination Specific Modelling in HAbitats Related to Space; http://www.biosmhars.eu/) is the first joint EU-Russia research project to address this issue. The first phase of this project aimed at developing, calibrating, and validating a mathematical model to predict the dispersion of microbial bio-aerosols in the BIOS facility (http://www.biosmhars.eu/expe/bios-3), a closed environment in size and concept relevant for space, so far without human activity and under Earth conditions. The long-term objective of the BIOSMHARS team is to develop a versatile and robust modeling tool to predict airborne microbial contaminant dispersion and deposition in a manned spacecraft in flight.

Microbes are intimately associated with life and biological processes on Earth and will always be present in manned space habitats. If a better understanding can be obtained on the dynamics of microbial dispersal, survival, and proliferation in indoor confined habitats, it will help to define better strategies to monitor, manage, and control microflora to benefit crew health and performance.

## Abundance of bacteria and fungi in the ISS

Each space agency has been continuously monitoring the abundance and diversity of bacteria and fungi in their respective modules in the ISS to better understand microbial dynamics in crewed habitats in space.

NASA previously reported microbial abundance in the ISS based on the continuous monitoring and recovery of viable cells using cultivation based methods (9). *Staphylococcus*, *Bacillus*, and *Micrococcus* species have been the most frequently recovered bacterial genera from air and surface sampling from the ISS based on quarterly samples returned between August 1998 and August 2011. The most commonly isolated fungal genera from the air and surface samples during the same time period were *Penicillium*, *Aspergillus*, *Cladosporium*, and *Hyphomycetes*. The most commonly isolated organisms from spaceflight water samples analyzed during ground identification between 2009 and 2012 were *Ralstonia pickettii* and *Burkholderia multivorans*.

In the United States Operating Segment of the ISS, the Water Recovery System (WRS) is a physicochemical system comprised of a Urine Processor Assembly (UPA) and Water Processor Assembly (WPA) that is designed to recycle crew member urine and humidity condensate for reuse as potable water. Direct counts by microscopic enumeration revealed 8.4 × 10^4^ cells mL^−1^ in the humidity condensate sample, but no colony-forming cells. In contrast, 3.3 × 10^5^ cells mL^−1^ were detected in a surface swab of the WRS waste tank, and included colony-forming bacteria and fungi recovered after a 12-d incubation on solid agar media. Based on 18S rRNA sequencing and phenotypic characterization, a fungal biofilm raft recovered from the filter was determined to be *Lecythophora mutabilis*. A bacterial isolate recovered from a biofouling sample of the membrane in the WRS was identified by 16S rRNA gene sequence data as *Methylobacterium radiotolerans* (unpublished data).

JAXA has also been continuously performing bacterial monitoring in Kibo (docked with the ISS in March 2008) since 2009 (Research title: Microbe). Sampling was performed in September 2009 (Microbe-I), October 2010 (Microbe-II), February 2011 (Microbe-II’), and October 2012 (Microbe-III). In this research, the relative abundance and phylogenetic affiliation of bacteria and fungi collected from the interior surfaces of Kibo were determined by quantitative PCR, PCR-DGGE, and the assembly of clone libraries. The surface of the Cell Biology Experiment Facility (CBEF; incubator), inside of the door of the CBEF, laptop palm rest, air intake, air diffuser, and handrail were selected as sampling sites. A new sampling device, the microbe-collecting adhesive sheet (Fig. 2), was used in addition to the traditional swabbing method (24).

Bacterial abundance at each site, except for the air intake, was equivalent to or less than the quantification limit of quantitative PCR (cells cm^−2^) in multiple experiments conducted in different missions (Microbe-I and Microbe-II). Meanwhile, their abundance was below the minimum detection limit at all sampling sites in Microbe-II’ and Microbe-III. The phylogenetic affiliation of bacteria collected from the Microbe-II mission was determined, and bacteria in the phyla *Actinobacteria* and *Firmicutes* were frequently detected on the surface of the PC palm rest and handrail, which were touched frequently by astronauts. Most of these bacteria have been detected on human hands as part of the normal human skin microbiota; thus, bacterial cells may be transferred to the surface of Kibo via astronaut contact.

Staphylococci and enterococci are a part of the normal human flora and are, thus, commensal microorganisms. However, they can also be opportunistic pathogens that cause a wide range of diseases. Therefore, the antibiotic resistance of staphylococci and enterococci isolated from the ISS was determined. In a collection of ISS isolates from sampling campaigns between 2002 and 2006, twenty-nine *Staphylococcus* and *Enterococcus* isolates were investigated for antibiotic resistance, horizontal transfer capability, and biofilm formation (46). Resistance to one or more antibiotics was detected in 22 out of 29 (75.8%) strains examined. The most prevalent resistance determinants among the staphylococci were *ermC*, *tetK*, and different *cat* genes conferring resistance to macrolide, tetracycline, and chloramphenicol, respectively (46). Plasmids were present in 86.2% of the isolates; eight of the *Enterococcus faecalis* isolates harbored a large plasmid of approximately 130 kb, likely to be self-transmissible. Transfer genes encoding key proteins of the conjugative transfer process, such as the ATPase delivering energy for the transfer process and the coupling protein indispensable for linking the DNA transfer and replication complex (Dtr) with the so-called mating pair formation (Mpf) complex from the well-known resistance plasmids from gram-positive bacteria such as pIP501, pRE25, pSK41, pGO1 and pT181, were detected in the total DNA of 86.2% of the strains. Most pSK41-homologous transfer genes were detected in isolates belonging to coagulase-negative staphylococci. Twenty-eight percent of the isolates contained at least one *vir* signature gene, *virB1*, encoding a muramidase locally opening the peptidoglycan in the cell wall, *virB4*, encoding the motor ATPase of the plasmid transfer process, and *virD4*, encoding the conjugative coupling protein, respectively. Through solid surface matings, it was demonstrated that several *Staphylococcus* spp. isolates could transfer their resistance genes to distinct *Enterococcus* and *Staphylococcus* spp. Biofilm formation was observed in 83% of the isolates (46). As methicillin-resistant *Staphylococcus aureus* (MRSA) or vancomycin-resistant *Enterococcus* (VRE) was not detected, the health risk associated with *Enterococcus* and *Staphylococcus* infections for the crew was assessed as low.

To monitor fungal microbiota in the ISS-Kibo, culture-based methods were used for swab- and flat sheet media samples in the Microbe-I experiment. Tests on orbital samples using either sample collection method were negative. Although adhesive sheet samples were also examined by field emission-scanning electron microscopy, no microbial cells were detected. However, fungal DNAs were detected using real-time PCR (33) and then analyzed by the clone library method. *Alternaria* sp. was the dominant fungal species before the launch, but the most abundant fungal species changed to *Malassezia* spp. on orbit. The dominant species found in ground control samples collected from the air conditioner diffuser, lab bench, door push panel, and facility surfaces in a university laboratory were *Inonotus* sp., *Cladosporium* sp., *Malassezia* spp., and *Pezicula* sp., respectively. *Malassezia* spp. constitute human skin microbiota and *Inonotus* sp., *Cladosporium* sp., and *Pezicula* sp. are often found in natural environments such as soil and plants. These results suggested that the fungal biota in Kibo changed from soil-borne fungi to a human origin. It was concluded in 2009 that cleanliness in Kibo was equivalent to that in an ISO Class 7 clean room environment on the ground (45).

In the Microbe-II experiment, 14 strains belonging to 5 fungal species grew on board Kibo and changes in the fungal community structure were observed using clone libraries (unpublished data). In the Microbe-III experiment (September to October, 2012), 10 strains belonging to 4 fungal species were cultured from surface samples, but no fungi grew from air samples collected by an air-sampling device.

A major source of the microbes found on surfaces in the ISS is thought to have been the human skin microbiota. The human skin microbiota includes various types of microorganisms, such as viruses, bacteria, and fungi. The body sites involved include sebaceous or oily sites, moist sites, and dry sites. The predominant bacterial species differ at each body site. For example, *Actinobacteria*, including *Corynebacterium* and *Propionibacterium* species, predominate at sebaceous or oily sites, glabellar creases, and canals (22). Regarding the fungal microbiota, *Malassezia* is the predominant group at all body sites, although a few hundred fungi exist on human skin (20, 48). Therefore, the fungal microbiota differs from the bacterial microbiota.

**Fig. 3:**
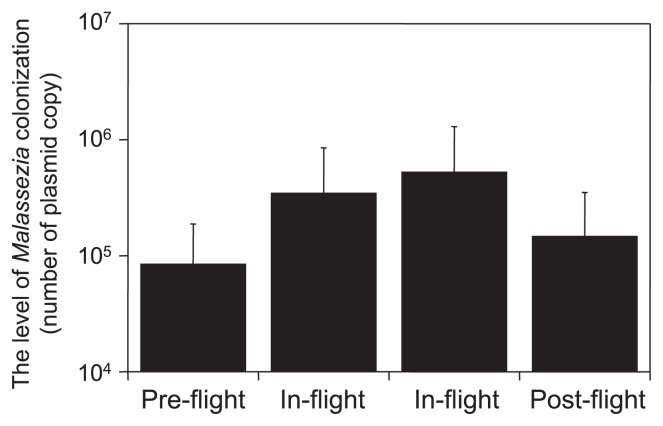
Temporal change in the colonization level of *Malassezia* in cheek skin samples from astronauts. The colonization level of *Malassezia* was determined using qPCR. Values show the average + standard deviation. Scale samples were collected before the visit to the ISS (pre-flight), during the stay in the ISS, and the return to earth (postflight).

The fungal microbiota of 10 astronauts who spent time on the ISS was analyzed by quantitative PCR. Skin samples were collected from the astronauts once during the pre-flight stage, twice during the in-flight stage, and once during the post-flight stage. The astronauts stayed on the ISS for 6 months. On the ISS, the colonization levels of *Malassezia* on the astronauts increased, but decreased in post-flight (Fig. 3) (unpublished data). A few hundred thousand high-quality reads were obtained by pyrosequencing, and more than100 fungal genera were detected in the samples collected from the 10 astronauts; *Cladosporium* and *Malassezia* were predominant, followed by *Aspergillus* and *Cryptococcus*. The fungal diversity of samples collected in-flight decreased while the fungal diversity of post-flight samples increased. The fungal microbiota sampled from both environmental surfaces in the ISS and skin of crew members revealed an increase in the proportion of *Malassezia* species. In cheek samples, *Malassezia* accounted for 95% of the overall fungal species in the in-flight samples, whereas it comprised only 50 and 60% of the pre- and post-flight samples, respectively (unpublished data). The level of microbial colonization markedly increased when access to bathing facilities was limited over a long period of time and the skin microbiota of astronauts may have changed for that reason.

From microbial abundance and their phylogenetic affiliation, the Kibo module has been microbiologically well maintained. However, increases were reported in bacterial and fungal numbers in previous space stations due to long-duration habitations (37), and microbial abundance in Kibo may also increase with prolonged exposure to astronauts. Continuous bacterial monitoring in Kibo is required to ensure crew safety and better understanding of microbial dynamics in space habitation environments.

## Microbial responses to microgravity

The short generation time of microorganisms makes them uniquely suited for studies to assess responses to altered environmental conditions. Therefore, microbial cells were among the first Earth-based life forms to be sent into the environment of space. Early studies revealed that the spaceflight environment did not affect the viability of microorganisms (28, 63, 64). However, by assessing *Escherichia coli* on several Vostok (Russian spacecraft) flights, these initial spaceflight studies documented an increase in the number of phage particles produced, as well as a variant colony type of the bacteria, which were concluded to be the result of spaceflight factors (28, 63, 64). The studies that followed included assessments of basic microbial characteristics, such as alterations in cell density as a result of spaceflight cultures. Over the course of several flight experiments, *S.* Typhimurium, *E. coli*, *Candida albicans*, and *Bacillus subtilis* displayed greater growth profiles in spaceflight than ground controls (12, 16, 25, 26, 49).

With evidence that bacteria were able to sense and respond to the microgravity environment of spaceflight, the concern of spaceflight researchers shifted to how these variations could impact on human health. Over the course of numerous spaceflights, researchers from various countries analyzed changes in antibiotic resistance by *E. coli* and *S. aureus* (51). Specifically, the minimal inhibitory concentrations (MIC) of oxacillin, chloramphenicol, and erythromycin for *S. aureus* and colistin and kanamycin for *E. coli* were evaluated among in-flight cultures and compared to ground controls. These studies reported an increase in bacterial resistance to all antibiotics tested for both *S. aureus* and *E. coli*. Additionally, the researchers observed a thickening of the cell wall that accompanied the increase in the antibiotic resistance of *S. aureus* once returned from flight (51).

To further define the impact of spaceflight culture on the pathogenesis of microorganisms, researchers again turned to *S.* Typhimurium, launching it into orbit, allowing it to grow, and returning it for assessments in a murine model of salmonellosis (59). Mice infected with bacteria cultured in-flight displayed a decreased time-to-death, increased percent mortality, and decrease in the lethal dose required to kill 50% of the mice (LD50) (59). In addition, samples that were fixed in-flight and analyzed on the ground revealed the differential expression of 167 genes and 73 proteins, which led to the identification of a possible role for the regulatory protein, Hfq, in the bacterial response to cultures in the spaceflight environment (59). This was the first study to elucidate both the molecular response connected with a regulatory mechanism and alterations in bacterial virulence as a consequence of growth in spaceflight. A follow-up investigation with *S.* Typhimurium confirmed these virulence findings and determined that the media ion concentration influenced the spaceflight-related virulence response in this organism (60). The transcriptional and proteomic responses of spaceflight cultured *Pseudomonas aeruginosa* have recently been documented, and the involvement of Hfq and the Hfq regulon was noted (15). These studies on the involvement of Hfq represent the first account of a common molecular regulatory mechanism, shared among different bacterial species, in response to the spaceflight environment. Although inroads into the mechanism(s) behind microbial adaptations have been made using spaceflight as a platform, and even though there have been more than 100 spaceflight experiments involving microorganisms during the past 50 years, our knowledge of how their response to this environmental parameter impacts their ability to cope with antibiotics and other environmental stresses has largely been generated through investigations utilizing a spaceflight analog cell culture system.

**Fig. 4:**
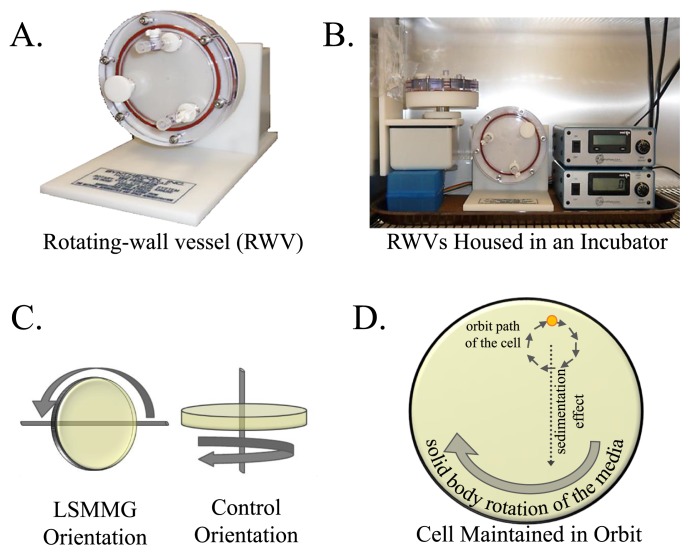
The Rotating-Wall Vessel (RWV) Bioreactor. (A and B) Image of the NASA-designed RWV apparatus. (C) The altered positioning of the RWV resulting in two culture orientations, the arrows depict the directions of rotation. The low-shear modeled microgravity (LSMMG) environment is achieved by rotation of the RWV on an axis parallel to the ground, whereas the axis of rotation in the control orientation is perpendicular to the ground. (D) Depiction of the orbital path of a cell when cultured in the LSMMG orientation. The continued combination of the sedimentation effect, whereby gravity and a lack of motility cause a cell to settle to the bottom of the vessel, and the clock-wise solid body rotation of the media results in continuous suspension of the cell in an orbit. Modified from Castro, *et al.*, 2011 (8).

Continuous microgravity conditions cannot be created on Earth; however, aspects of the microgravity environment can be mimicked using ground-based simulators. The rotating-wall vessel (RWV) bioreactor has been increasingly used to enhance our understanding of microbial responses that may be occurring during spaceflight (8, 18, 30, 34–36). The RWV (Fig. 4A) is an optimized form of suspension culture in which cells are grown under physiologically relevant low-fluid-shear conditions. A cell in liquid media in microgravity is known to experience two unique aspects that are important for modeling this environment: 1) remaining in a constant state of suspension and 2) experiencing a quiescent surrounding, devoid of shearing, turbulent forces. It is these aspects of the microgravity culture environment that the RWV bioreactor effectively simulates. The components of the RWV bioreactor system include the vessel, rotation base unit with an oxygen pump, and power supply. The vessel is a thin, cylindrical disc to which the cell culture media is introduced by a syringe via ports on the face of the vessel. Once attached to the base unit, the power supply initiates rotation of the vessel and provides a supply of oxygen through a gas permeable membrane on the inner backside of the vessel. The entire system can be housed in an incubator to allow for optimal cell growth at a fixed temperature (Fig. 4B). As the rotation of the fully filled vessel increases, its rotational velocity is transferred radially inward until relative fluid motion ceases (Fig. 4C), at which point the solid body rotation of the fluid is achieved (27). A cell within this environment experiences the sedimentation effect imparted by gravity. As it begins to fall toward the bottom of the vessel (“settle out”), it is carried back upward by the solid body rotation of the media and, thus, remains suspended in the fluid in an orbital path (Fig. 4D), modeling the first aspect of the microgravity environment described above. Therefore, when cultured in the RWV bioreactor, a microorganism experiences a quiescent, low-shear, low-turbulent environment that is devoid of shearing forces and, thus, is analogous to the second aspect of spaceflight described above. As it is important to note the low-shear effects of the fluid on cells, the term Low-Shear Modeled Microgravity (LSMMG) has been adopted for use to accurately describe the environment produced by the RWV bioreactor (57).

Through the study of numerous microorganisms cultured within the RWV bioreactor, similarities have been noted to both spaceflight responses and among organisms (36). For example, scanning electron microscopy images revealed an unidentified extracellular matrix around *S.* Typhimurium cells following spaceflight cultures (59). Additionally, biofilm formation in *P. aeruginosa* was documented during growth in spaceflight (32). In response to the modeled microgravity conditions within the RWV bioreactor, biofilm formation by *P. aeruginosa*, *S. aureus*, *E. coli*, and *C. albicans* was increased (8, 14, 31, 47). This increase in biofilm formation has been correlated with an increase in resistance by *S. aureus*, *E. coli*, and *C. albicans*. Another significant commonality between organisms is changes in responses to environmental stressors following cultivation within the RWV. In response to a challenge with osmotic stress, *S.* Typhimurium and *E. coli* each displayed increased resistance following cultivation within the RWV (31, 58). Furthermore, *S.* Typhimurium, *E. coli*, and *P. aeruginosa* all exhibited an increased ability to survive thermal conditions post-RWV culture (2, 14, 31, 58). However, increased survivability to environmental stress has not always correlated to RWV culture conditions. While *E. coli* and *P. aeruginosa* are better able to withstand oxidative stress, *S.* Typhimurium and *S. aureus* were found to be more sensitive to this stressor following growth within the RWV (2, 8, 14, 58). A summary of certain bacterial, fungal, and archaeal responses to the simulated microgravity conditions within the RWV bioreactors can be found in Table 3.

Microgravity is merely one unique environmental parameter of the spaceflight environment. Investigations assessing the responses of microorganisms to additional factors, such as radiation and the vacuum of space, have been conducted in space and through simulated laboratory experimentation (23). The findings of these studies showed that organisms and bacterial spores were able to withstand the extremes associated with exposure to the environment of space (23). Specifically, the bipolar lichen species, *Rhizocarpon geographicum* and *Xanthoria elegans* isolated at above 2,000 m from the mountains of Spain, were able to survive 16 d of exposure to radiation and the vacuum of space (44). Assessments of the survival and DNA repair mechanism(s) utilized by microorganisms to withstand these extremes are ongoing. A thorough discussion of microbial responses to the numerous environmental parameters associated with the spaceflight environment can be found within the review by Horneck *et al.* (23).

## New methods for microbial monitoring in space habitats

As mentioned above, the cleanliness of the spacecraft and crewed environments has been a focus of each space agency since their inception. The historical records of microbial monitoring of the air, water, and surfaces of the spacecraft have provided an insight into the diversity and abundance of microorganisms within the spacecraft environment.

The current methods of bacterial and fungal monitoring on the ISS depend on the culturing of microorganisms during spaceflight and subsequent ground-based identification. This approach requires substantial crew time and uses perishable consumables that require frequent resupply because of short shelf lives. These resource requirements in combination with sample collection and analytical constraints limit rapid responses to microbial contamination when detected. Furthermore, sample return and ground-based identification will not be an option during future long-term missions. Previous environmental monitoring using traditional culture-based monitoring methods has resulted in an understanding of the culturable microbiota of the spacecraft, but this only represents a small percentage of the extant microbial community. As we move farther away from Earth and deeper into the frontier of space, the development of new microbial monitoring technologies aimed at detecting, quantifying, and identifying the presence of target organisms of interest will become increasingly important.

As human spaceflight moves beyond a low-Earth orbit for extended periods of time, the ability to monitor microbial contamination in-flight is just as critical as the need to rapidly detect, diagnose, and treat infectious diseases. NASA is currently working to define existing and develop new microbial detection methods that will provide spaceflight crews with a rapid, simple, and an autonomous means of detecting microorganisms in the spaceflight environment, thereby mitigating microbial risks to both crew and craft. Subject matter experts from industry and academia, in cooperation with NASA microbiologists, generated a list of recommendations for long-duration mission microbial monitoring during a 2011 workshop at the NASA Johnson Space Center. The consensus of the group was that NASA should investigate and implement a molecular-based form of microbial detection, such as real-time PCR, that could be performed in-flight. Moving forward, NASA scientists initiated a comprehensive market survey of commercially available real-time PCR platforms. In order to be considered for flight evaluation, the technology must meet multiple design criteria:

1. Use quantitative or semi-quantitative PCR technology with multiplexing abilities;
2. Have a low limit of detection (≤ 400 cells sample^−1^ in a 100 μL sample volume);
3. Use reagents that are shelf-stable at room temperature for a minimum of six months;
4. Provide a rapid assessment of the microbial environment on the ISS;
5. Expand monitoring of microorganisms beyond current means;
6. Reduce the frequency of sampling events;
7. Allow for crew autonomy during sample analysis; and
8. Have the ability to function in a microgravity environment.

Three platforms were selected for testing: the Cepheid SmartCycler, BioFire Diagnostic (formerly Idaho Technologies, Inc.) RAZOR EX, and iCubate 2.0 systems. Testing is ongoing and these instruments are being validated to assess their ability to detect target pathogens in potable water. The RAZOR EX and iCubate 2.0 systems have demonstrated the ability to meet low detection limits using a sample-to-answer methodology. To fairly identify and provide a screening process for additional hardware that may be able to meet the needs of microbial monitoring during flight, a testing matrix was developed during initial testing for further evaluations of existing commercial technology and the identification of additional requirements to be used during a competitive proposal process.

In addition to molecular-based identification methods, NASA is also investigating simple portable systems for microbial monitoring. For example, technology utilizing gold nanoparticles for the detection of medically significant microorganisms is of interest because gold nanoparticles have several properties, *e.g.* high stability, low toxicity, and photonic properties, that support their use in biodetection applications for crewed habitats in space (50). The absorption of light is correlated to nanoparticle size, and therefore, the reflected color of the nanoparticles changes as a result of the size of the particle and/or aggregation of particles. In addition to their optical properties, gold nanoparticles can be functionalized by the covalent attachment of various biomolecules. The functionalization process has provided targeted delivery of these nanoparticles to numerous cell types, increased their detection by bioimaging, and improved their utility for gene and drug delivery for multiple therapeutic and diagnostic applications (10). The sensitivity and specificity of the assay is dependent on the use of different ligands (*i.e.*, passivating molecules) on the surface of the gold clusters. While the physical chemical nature of the passivating layer is normally to simply stabilize the gold core, an appropriate modification of this layer can lead to “sensing” of particular functional groups on the target. Therefore, gold nanoparticles functionalized by the conjugation of a ligand can serve as chromogenic biosensors in which binding of the target to the ligand results in the agglomeration of nanoparticles and a red-to-purple shift.

In proof-of-concept studies of the gold nanoparticle approach, NASA researchers are developing a biodetection system to detect *S. aureus*, a common infectious agent that has been repeatedly isolated aboard the ISS (10, 40). Gold nanoparticles have been modified by the covalent attachment of an antibody that has a high binding affinity for a protein on the cell wall of *S. aureus*. Data from preliminary testing of the gold nanoparticle biodetection system has shown that *S. aureus* could be detected in a sample in as quickly as 10 min. The system, as constructed, was specific to *S. aureus*, as other bacteria assessed during the studies were not bound by the nanoparticles, and the binding of *S. aureus* was not affected by the presence of other microorganisms in the sample. These initial investigations have shown that a simple system capable of rapidly detecting a low-density target pathogen in a complex environment using simple to interpret colorimetric indication is feasible. The development and validation of this system is ongoing.

Culture-independent techniques using direct fluorescent staining are also effective for the rapid detection of microbes. However, these methods are often labor intensive and cell concentration steps or large sample volumes are required to obtain reliable results when samples contain low numbers of target bacteria. Flow cytometry is an effective alternative to fluorescence microscopy because the procedure is rapid and sensitive. However, flow cytometers are often complex and sometimes require skilled operators for their operation, maintenance, and analytical interpretation. Therefore, a simpler and smaller system should be more useful for “on-board” counting of targeted microorganisms in a space habitat.

**Fig. 5:**
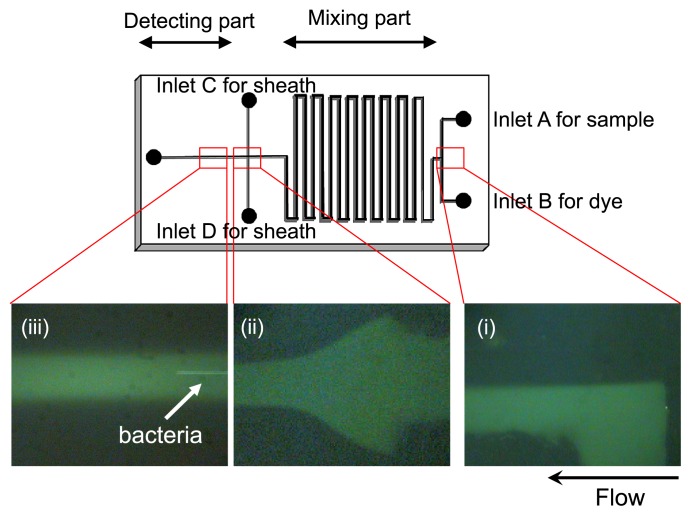
Details of the microfluidic device for on-chip staining and counting of bacterial cells (size: 5 cm × 2.5 cm). (i) Samples and fluorescent dye solution flow separately and are then mixed through the “mixing part” of the microchannel. (ii) Alignment of sample flow by sheath fluid. (iii) Flow of bacterial cells in the “detecting part” of the microchannel.

Microfluidic systems that use microfluidic channels (*e.g.*, microchips [Fig. 5]) or capillaries are powerful tools for “on-chip” fluorescent staining and enumeration of targeted microbial cells in any sample that can be fluidized (62). Microchip-based analyses are faster, can be performed on very small sample scales, and consume less sample and reagent volumes than conventional approaches (5). Furthermore, microfluidic devices can reduce the biohazard risk because cells are analyzed in a closed system using disposable components or devices that can be easily cleaned of contaminants and sterilized after each use. Therefore, microfluidic devices in various forms have been used to miniaturize flow cytometers (on-chip flow cytometry [19, 21, 43, 56]). However, most of these microfluidic devices were developed to entrap or analyze the characteristics of targeted cells rather than to determine cell numbers by a simple procedure. A new microfluidic system was recently developed to count total bacteria and harmful bacteria without extensive sample preparation steps such as sample concentration or the pre-staining of bacterial cells (Fig. 5).

This new system has been applied to count bacterial cells in potable water. Bacteria in potable water samples were stained “on-chip” with the DNA-staining dye (4′,6-diamidino-2- phenylindole [DAPI]) and counted in a microfluidic system for direct comparisons against bacterial enumeration determined by fluorescence microscopy (62). The ratios of the microfluidic counts to microscopic counts were 73% in purified household tap water and 80% in groundwater samples. The microfluidic system can detect bacterial cells in potable freshwater within one hour and the system enables the rapid detection of significant increases in bacterial numbers in water samples, a critical requirement for the routine evaluation of microbiological water quality on orbit.

JAXA is now developing a portable microfluidic system for “real-time” and “on-board” microbial monitoring. This system will contribute to safe and sustainable long-duration space habitation. In addition, these rapid microbiological systems will not require the cultivation of potentially pathogenic microbes in the closed ecological system of the spacecraft and will enable the rapid generation of highly accurate results. In addition to their application in space, these methods can be used in various industries such as pharmaceutical manufacturing, food production, and as point-of-care diagnostic techniques in clinical settings.

## Future perspective of “environmental microbiology in crewed habitats in space”

As defined by the roadmaps of each space agency, indoor environmental quality control is critical in order to reduce potential hazards for the crew and vehicle. On-going microbiological monitoring in the ISS must be continued, improved, and expanded to accumulate original data on microbial dynamics in the space station environment, and microbial spaceflight experiments to predict microbial dynamics in crewed habitats in space should be promoted. Evaluating data on microbial community dynamics in the ISS can be promoted by formally sharing currently collected information among the international partners, then defining and verifying a common standardization of the protocols for microbiological monitoring in the space station environment for future monitoring. With better data sharing and protocol standardization, we can define upper and lower thresholds for the indoor environmental quality control of air, water, and surfaces in space habitats.

## Figures and Tables

**Table 1:** NASA current microbial monitoring

Sample Type	Media	Incubation Temp/Time
Pre-flight Air^1^	TSA^5^ and SDA^6^ w/chloramphenicol plates	TSA: incubation at 37°C for 48 h SDA: incubation at 30°C for 5 d
Flight Air^2^	TSA and SDA w/chloramphenicol plates	TSA and SDA plates incubated at ambient cabin temperature for 5 d
Pre-flight Surface	Sterile nuclease-free water-moistened swab into 3 mL of TSB^7^. 0.1 mL each spread onto TSA (4 plates + 2 plates 0.01 mL) and SDA (2 plates), SDA w/chloramphenicol (1 plate), PDA^8^ (1 plate)	TSA incubated at 35°C±2°C for 48±2 h SDA 30°C±2°C for 5 d
Flight Surface	Collection using either a sterile saline-moistened swab onto a contact slide or direct sampling using a contact slide depending on the location	Contact slides (TSA or SDA w/chloramphenicol) incubated at ambient cabin temperature for 5 d
Pre-flight Water^3^,^4^	R2A Milli-flex cassettes	R2A incubated at 35°C±2°C for 48±2 h
Flight Water	Microbial Capture Device with modified R3A broth; Coliform detection using Colisure Reagent	Microbial Capture Device and Coliform Detection bags incubated at ambient cabin temperature for 44±4 h

1 SAS Super 180 Air sampler collecting for 1 min at 180 L min^−1^.

2 Modified portable impaction sampler (Burkard Manufacturing Co. Ltd., Hertfordshire, UK); 84.9 L sample collected.

3 Processed by filtration for heterotrophic plate counts and molecular identification.

4 the only water samples currently obtained are typically courtesy samples shared by ESA or JAXA.

5 TSA: Trypticase Soy Agar,

6 SDA: Sabouraud dextrose agar,

7 TSB: Trypticase Soy Broth,

8 PDA: potato dextrose agar

**Table 2:** NASA preflight and inflight acceptability limits for air, water, and surface samples

Sample	Air	Surface	Water
Preflight	300 CFU m^−3^ Bacteria	500 CFU 100 cm^−2^ Bacteria	50 CFU mL^−1^
	50 CFU m^−3^ Fungi	10 CFU 100 cm^−2^ Fungi	No detectable coliforms
Inflight	1,000 CFU m^−3^ Bacteria	10,000 CFU 100 cm^−2^ Bacteria	50 CFU mL^−1^
	100 CFU m^−3^ Fungi	100 CFU 100 cm^−2^ Fungi	No detectable coliforms

**Table 3:** Microbial responses to modeled microgravity

Microorganism	Response to Modeled Microgravity within the RWV bioreactor	Reference
*S.* Typhimurium χ^3339^	Increased virulence in a mouse model, resistance to acid, thermal, and osmotic stresses, and macrophage survival Decreased LPS production, resistance to oxidative stress, and Hfq expression Differential gene expression	Nickerson, 2000 (35) Wilson, 2002 (57) Wilson, 2002 (58) Wilson, 2007 (59)
*S.* Typhimurium 14028	Increased virulence in a mouse model and cellular invasion Differential gene expression	Chopra, 2006 (11)
*E. coli* AMS6	Increased biofilm formation and resistance to osmotic, ethanol, and antibiotic stresses	Lynch, 2006 (31)
*E. coli* E2348/69	Increased intimin production	Carvalho, 2005 (7)
*E. coli* MG1655	Decreased growth Differential gene expression	Tucker, 2007 (52)
*E. coli* K12	Differential gene expression	Vukanti, 2008 (54)
*E. coli* O83:H1	Increased resistance to thermal and oxidative stresses and adhesion to epithelial cells	Allen, 2008 (2)
*P. aeruginosa* PAO1	Increased biofilm formation, elastase, rhamnolipid, and alginate production; resistance to oxidative and thermal stress, and Hfq expression Differential gene expression	Crabbe, 2008 (13) Crabbe, 2010 (14)
*Streptococcus pneumoniae* TIGR4	Differential gene expression	Allen, 2006 (1)
*S. aureus* N315	Increased biofilm formation and susceptibility to whole blood Decreased growth, carotenoid production, resistance to oxidative stress, and Hfq expression	Castro, 2011 (8)
*S. aureus* RF1, RF6, RF11	Decreased carotenoid production and hemolytic activity Differential gene expression	Rosado, 2010 (42)
*S. aureus* 25923	Increased growth and membrane integrity	Vukanti, 2012 (55)
*Stenotrophomonas paucimobilis* 10829	Decreased growth	Baker, 2005 (4)
*S. paucimobilis* isolated from the ISS water system	Increased growth	Baker, 2005 (4)
*Yersinia pestis* KIMD27	Decreased Hela cell rounding	Lawal, 2010 (29)
*Haloferax mediterranei*	Increased antibiotic resistance Differential pigment production and protein expression	Dornmayr-Pfaffenhuemer, 2011 (17)
*Halococcus dombrowskii*	Decreased cell aggregation Differential pigment production and protein expression	Dornmayr-Pfaffenhuemer, 2011 (17)
*Saccharomyces cerevisiae*	Increased aberrant budding Differential gene expression	Purevdorj-Gage, 2006 (41)
*Candida albicans*	Increased filamentous growth, biofilm formation, antimicrobial resistance Differential gene expression	Altenburg, 2008 (3) Searles, 2011 (39)

## References

[1] Allen CA, Galindo CL, Pandya U, Watson DA, Chopra AK, Niesel DW (2006). Transcription profiles of *Streptococcus pneumoniae* grown under different conditions of normal gravitation. Acta Astronautica.

[2] Allen CA, Niesel DW, Torres AG (2008). The effects of low-shear stress on adherent-invasive *Escherichia coli*. Environ Microbiol.

[3] Altenburg SD, Nielsen-Preiss SM, Hyman LE (2008). Increased filamentous growth of *Candida albicans* in simulated microgravity. Genom Proteom Bioinform.

[4] Baker PW, Leff L (2004). The effect of simulated microgravity on bacteria from the Mir space station. Microgravity Sci Technol.

[5] Blankenstein G, Larsen UD (1998). Modular concept of a laboratory on a chip for chemical and biochemical analysis. Biosens Bioelectron.

[6] Borchers AT, Keen CL, Gershwin ME (2002). Microgravity and immune responsiveness: Implications for space travel. Nutrition.

[7] Carvalho HM, Teel LD, Goping G, O’Brien AD (2005). A three-dimensional tissue culture model for the study of attach and efface lesion formation by enteropathogenic and enterohaemorrhagic *Escherichia coli*. Cell Microbiol.

[8] Castro SL, Nelman-Gonzalez M, Nickerson CA, Ott CM (2011). Induction of attachment-independent biofilm formation and repression of Hfq expression by low-fluid-shear culture of *Staphylococcus aureus*. Appl Environ Microbiol.

[9] Castro VA, Thrasher AN, Healy M, Ott CM, Pierson DL (2004). Microbial characterization during the early habitation of the International Space Station. Microb Ecol.

[10] Chan PH, Chen YC (2012). Human serum albumin stabilized gold nanoclusters as selective luminescent probes for *Staphylococcus aureus* and methicillin-resistant *Staphylococcus aureus*. Anal Chem.

[11] Chopra V, Fadl AA, Sha J, Chopra S, Galindo CL, Chopra AK (2006). Alterations in the virulence potential of enteric pathogens and bacterial-host cell interactions under simulated microgravity conditions. J Toxicol Environ Health A.

[12] Ciferi O, Tiboni O, Orlandoni AM, Marchesi ML, Longdon N, David V (1988). The effects of microgravity on genetic recombination in *Escherichia coli*. Biorack on Spacelab D1: An Overview of the First Flight of Biorack, an ESA Facility for Life Sciences Research in Microgravity.

[13] Crabbe A, de Boever P, Van Houdt R, Moors H, Mergeay M, Cornelis P (2008). Use of the rotating wall vessel technology to study the effect of shear stress on growth behaviour of *Pseudomonas aeruginosa* PA01. Environ Microbiol.

[14] Crabbe A, Pycke B, Van Houdt R, Monsieurs P, Nickerson C, Leys N, Cornelis P (2010). Response of *Pseudomonas aeruginosa* PAO1 to low shear modelled microgravity involves AlgU regulation. Environ Microbiol.

[15] Crabbe A, Schurr MJ, Monsieurs P (2011). Transcriptional and proteomic responses of *Pseudomonas aeruginosa* PAO1 to spaceflight conditions involve Hfq regulation and reveal a role for oxygen. Appl Environ Microbiol.

[16] Crabbe A, Nielsen-Preiss SM, Woolley CM (2013). Spaceflight enhances cell aggregation and random budding in *Candida albicans*. PLOS ONE.

[17] Dornmayr-Pfaffenhuemer M, Legat A, Schwimbersky K, Fendrihan S, Stan-Lotter H (2011). Responses of haloarchaea to simulated microgravity. Astrobiology.

[18] Fang A, Pierson DL, Mishra SK, Koenig DW, Demain AL (1997). Gramicidin S production by *Bacillus brevis* in simulated microgravity. Curr Microbiol.

[19] Fu AY, Chou HP, Spence C, Arnold FH, Quake SR (2002). An integrated microfabricated cell sorter. Anal Chem.

[20] Gao Z, Perez-Perez GI, Chen Y, Blaser MJ (2010). Quantitation of major human cutaneous bacterial and fungal populations. J Clin Microbiol.

[21] Gawad S, Schild L, Renaud P (2001). Micromachined impedance spectroscopy flow cytometer for cell analysis and particle sizing. Lab Chip.

[22] Grice EA, Segre JA (2011). The skin microbiome. Nat Rev Microbiol.

[23] Horneck G, Klaus DM, Mancinelli RL (2010). Space microbiology. Microbiol Mol Biol Rev.

[24] Ichijo T, Hieda H, Ishihara R, Yamaguchi N, Nasu M (2013). Bacterial monitoring with adhesive sheet in the International Space Station-“Kibo”, the Japanese Experiment Module. Microbes Environ.

[25] Kacena MA, Merrell GA, Manfredi B, Smith EE, Klaus DM, Todd P (1999). Bacterial growth in space flight: logistic growth curve parameters for *Escherichia coli* and *Bacillus subtilis*. Appl Microbiol Biotechnol.

[26] Klaus D, Simske S, Todd P, Stodieck L (1997). Investigation of space flight effects on *Escherichia coli* and a proposed model of underlying physical mechanisms. Microbiol.

[27] Klaus DM (2001). Clinostats and bioreactors. Gravit Space Biol Bull.

[28] Klemparskaya NN (1964). Effect of the conditions of cosmic flight on the dissociation of *Escherichia coli*. Artif Earth Satell.

[29] Lawal A, Jejelowo OA, Rosenzweig JA (2010). The effects of low-shear mechanical stress on *Yersinia pestis* virulence. Astrobiology.

[30] Lynch SV, Brodie EL, Matin A (2004). Role and regulation of σ^S^in general resistance conferred by low-shear simulated microgravity in *Escherichia coli*. J Bacteriol.

[31] Lynch SV, Mukundakrishnan K, Benoit MR, Ayyaswamy PS, Matin A (2006). *Escherichia coli* biofilms formed under low-shear modeled microgravity in a ground-based system. Appl Environ Microbiol.

[32] McLean RJ, Cassanto JM, Barnes MB, Joseph HK (2001). Bacterial biofilm formation under microgravity conditions. FEMS Microbiol Lett.

[33] Miyajima Y, Satoh K, Uchida T, Yamada T, Abe M, Watanabe S, Makimura M, Makimura K (2013). Rapid real-time diagnostic PCR for *Trichophyton rubrum* and *Trichophyton mentagrophytes* in patients with tinea unguium and tinea pedis using specific fluorescent probes. J Dermatol Sci.

[34] Nauman EA, Ott CM, Sander E, Tucker DL, Pierson D, Wilson JW, Nickerson CA (2007). Novel quantitative biosystem for modeling physiological fluid shear stress on cells. Appl Environ Microbiol.

[35] Nickerson CA, Ott CM, Mister SJ, Morrow BJ, Burns-Keliher L, Pierson DL (2000). Microgravity as a novel environmental signal affecting *Salmonella enterica* serovar Typhimurium virulence. Infect Immun.

[36] Nickerson CA, Ott CM, Wilson JW, Ramamurthy R, Pierson DL (2004). Microbial responses to microgravity and other low-shear environments. Microbiol Mol Biol Rev.

[37] Novikova ND (2004). Review of the knowledge of microbial contamination of the Russian manned spacecraft. Microb Ecol.

[38] Ott M, Pierson D, Shirakawa M, Tanigaki F, Hida M, Yamazaki T, Shimazu T, Ishioka N (2014). Space habitation and microbiology: status and roadmap of space agencies. Microbes Environ.

[39] Pierson D, Botkin DJ, Bruce RJ, Castro VA, Smith MJ, Oubre CM, Ott CM, Moldenhauer J (2012). Microbial Monitoring of the International Space Station. Environmental Monitoring: A Comprehensive Handbook.

[40] Pierson DL, Chidambaram M, Heath JD, Mallary L, Mishra SK, Sharma B, Weinstock GM (1996). Epidemiology of *Staphylococcus aureus* during space flight. FEMS Immunol Med Microbiol.

[41] Purevdorj-Gage B, Sheehan KB, Hyman LE (2006). Effects of low-shear modeled microgravity on cell function, gene expression, and phenotype in *Saccharomyces cerevisiae*. Appl Environ Microbiol.

[42] Rosado H, Doyle M, Taylor PW, Hinds J (2009). Low-shear modelled microgravity alters expression of virulence determinants of *Staphylococcus aureus*. Acta Astronautica.

[43] Sakamoto C, Yamaguchi N, Nasu M (2005). Rapid and simple quantification of bacterial cells by using a microfluidic device. Appl Environ Microbiol.

[44] Sancho LG, de la Torre R, Horneck G, Ascaso C, de Los Rios A, Pintado A, Wierzchos J, Schuster M (2007). Lichens survive in space: results from the 2005 LICHENS experiment. Astrobiology.

[45] Satoh K, Nishiyama Y, Yamazaki T, Sugita T, Tsukii Y, Takatori K, Benno Y, Makimura K (2011). Microbe-I: fungal biota analyses of the Japanese experimental module KIBO of the International Space Station before launch and after being in orbit for about 460 days. Microbiol Immunol.

[46] Schiwon K, Arends K, Rogowski KM, Fürch S, Prescha K, Sakinc T, Van Houdt R, Werner G, Grohmann E (2013). Comparison of antibiotic resistance, biofilm formation and conjugative transfer of *Staphylococcus* and *Enterococcus* isolates from International Space Station and Antarctic research station Concordia. Microb Ecol.

[47] Searles SC, Woolley CM, Petersen RA, Hyman LE, Nielsen-Preiss SM (2011). Modeled microgravity increases filamentation, biofilm formation, phenotypic switching, and antimicrobial resistance in *Candida albicans*. Astrobiology.

[48] Sugita T, Zhang E, Tanaka T, Nishikawa A, Tajima M, Tsuboi R (2013). Recent advances in research on *Malassezia* microbiota in humans. Med Mycol J.

[49] Taylor GR (1974). Space microbiology. Annu Rev Microbiol.

[50] Tiwari PM, Vig K, Dennis VA, Singh SR (2011). Functionalized gold nanoparticles and their biomedical applications. Nanomaterials.

[51] Tixador R, Richoilley G, Gasset G (1985). Preliminary results of Cytos 2 experiment. Acta Astronautica.

[52] Tucker DL, Ott CM, Huff S, Fofanov Y, Pierson DL, Willson RC, Fox GE (2007). Characterization of *Escherichia coli* MG1655 grown in a low-shear modeled microgravity environment. BMC Microbiol.

[53] Venkateswaran K, La Duc MT, Horneck G (2014). Microbial existence in controlled habitats and their resistance to space conditions. Microbes Environ.

[54] Vukanti R, Mintz E, Leff L (2008). Changes in gene expression of *E. coli* under conditions of modeled reduced gravity. Microgravity Sci Technol.

[55] Vukanti R, Model MA, Leff LG (2012). Effect of modeled reduced gravity conditions on bacterial morphology and physiology. BMC Microbiol.

[56] Wang Z, El-Ali J, Engelund M, Gotsaed T, Perch-Nielsen IR, Mogensen KB, Snakenborg D, Kutter JP, Wolff A (2004). Measurements of scattered light on a microchip flow cytometer with integrated polymer based optical elements. Lab Chip.

[57] Wilson JW, Ott CM, Ramamurthy R, Porwollik S, McClelland M, Pierson DL, Nickerson CA (2002). Low-Shear modeled microgravity alters the *Salmonella enterica* serovar Typhimurium stress response in an RpoS-independent manner. Appl Environ Microbiol.

[58] Wilson JW, Ramamurthy R, Porwollik S, McClelland M, Hammond T, Allen P, Ott CM, Pierson DL, Nickerson CA (2002). Microarray analysis identifies *Salmonella* genes belonging to the low-shear modeled microgravity regulon. Proc Natl Acad Sci USA.

[59] Wilson JW, Ott CM, Honer zu Bentrup K (2007). Space flight alters bacterial gene expression and virulence and reveals a role for global regulator Hfq. Proc Natl Acad Sci USA.

[60] Wilson JW, Ott CM, Quick L (2008). Media ion composition controls regulatory and virulence response of *Salmonella* in spaceflight. PLOS ONE.

[61] Yamaguchi N, Hieda H, Nasu M (2010). Simple and reliable swabbing procedure for monitoring microbes in the International Space Station. Eco Eng.

[62] Yamaguchi N, Torii M, Uebayashi Y, Nasu M (2011). Rapid, semiautomated quantification of bacterial cells in freshwater by using a microfluidic device for on-ship staining and counting. Appl Environ Microbiol.

[63] Zhukov-Verezhnikov NN, Mayskiy IN, Yazdovskiy VI, Pekhov AP, Rybakov NI, Gyurdzhian AA, Antipov VV (1962). Microbiological and cytological studies on spaceships. Probl Space Biol.

[64] Zhukov-Verezhnikov NN, Mayskiy IN, Yazdovskiy VI (1963). Problems of space microbiology and cytology. Probl Space Biol.

